# Surface, Subsurface and Tribological Properties of Ti6Al4V Alloy Shot Peened under Different Parameters

**DOI:** 10.3390/ma13194363

**Published:** 2020-09-30

**Authors:** Yasemin Yıldıran Avcu, Okan Yetik, Mert Guney, Eleftherios Iakovakis, Tamer Sınmazçelik, Egemen Avcu

**Affiliations:** 1 Department of Mechanical Engineering, Kocaeli University, Kocaeli 41001, Turkey; yaseminyildiran89@gmail.com (Y.Y.A.); oknyetik@gmail.com (O.Y.); tamersc@yahoo.com (T.S.); 2 School of Materials, The University of Manchester, Manchester M13 9PL, UK; 3 Department of Civil and Environmental Engineering, Nazarbayev University, Nur-Sultan 010000, Kazakhstan; mert.guney@nu.edu.kz; 4 The Environment and Resource Efficiency Cluster (EREC), Nazarbayev University, Nur-Sultan 010000, Kazakhstan; 5 Department of Mechanical Aerospace and Civil Engineering, The University of Manchester, Manchester M13 9PL, UK; eleftherios.iakovakis@manchester.ac.uk; 6 Ford Otosan Ihsaniye Automotive Vocational School, Kocaeli University, Kocaeli 41650, Turkey

**Keywords:** materials engineering, plastic deformation, shot peening, surface engineering, titanium (Ti) alloy, wear testing

## Abstract

Ti6Al4V alloy was shot peened by using stainless-steel shots with different sizes (0.09–0.14 mm (S10) and 0.7–1.0 mm (S60)) for two durations (5 and 15 min) using a custom-designed peening system. The shot size was the main parameter modifying the roughness (0.74 µm for S10 vs. 2.27 µm for S60), whereas a higher peening time slightly increased roughness. Hardness improved up to approximately 35% by peening with large shots, while peening time was insignificant in hardness improvement. However, longer peening duration with large shots led to an unwanted formation of micro-cracks and delamination on the peened surfaces. After dry sliding wear tests, the mass loss of peened samples (S60 for 15 min) was 25% higher than that of un-peened samples, while the coefficient of friction decreased by 12%. Plastically deformed regions and micro-scratches were observed on the worn surfaces, which corresponds to mostly adhesive and abrasive wear mechanisms. The present study sheds light on how surface, subsurface and tribological properties of Ti6Al4V vary with shot peening and peening parameters, which paves the way for the understanding of the mechanical, surface, and tribological behavior of shot peened Ti6Al4V used in both aerospace and biomedical applications.

## 1. Introduction

Titanium (Ti) alloys are widely used in implants [1,2], orthopaedic applications [3], chemical [4], automotive [5,6], and aerospace [7,8] industries owing to their high specific strength [9,10,11], corrosion resistance [6,12,13], non-toxicity [14,15], high biocompatibility [15], and fatigue [6] as well as formability [4]. However, they exhibit poor tribological properties [6,16] due to their low resistance to shear deformation [7], which limits their use in various applications [3,4]. For instance, some relatively poor tribological properties of Ti alloys (low abrasive wear resistance, low fretting wear resistance, and high coefficient of friction (CoF) [3,14,17,18]) limit their usage as hip replacements [19,20] and other artificial joint material [14,21]. Additionally, even a minor surface degradation of Ti alloys caused by contact may trigger catastrophic failure mechanisms such as fatigue and corrosion, which tend to start from previously damaged surface sites. Thus, understanding and improving surface features is vitally important to prevent potential catastrophic failure [7,22]. 

Shot peening has been widely used to improve the fatigue behavior of materials by retarding the formation and growth of cracks [23,24,25,26], changing the crystal structure [27,28,29,30], decreasing the grain size [7,16,31,32], increasing the density of dislocation [29,30], improving hardness [20,33,34], and inducing beneficial compressive residual stress [20,33]. More specifically, shot peening is a mechanical surface treatment mostly performed by the repeated impact of steel shots at high impact velocities onto surfaces of materials [34,35,36]. It has been commonly used in various industrial applications (e.g., biomedical [14], aerospace [37], and automotive industries [5,38]) owing to its ease of application and low process cost [34,39]. It is an effective surface treatment that improves the service life of components subjected to general fatigue as well as fretting fatigue by preventing crack initiation and propagation through improving the mechanical and physical properties [31,34]. Thus, the effects of shot peening in plain fatigue behavior have been previously investigated in detail [23,32,40,41,42,43,44,45]. By contrast, the modification of surface and subsurface properties such as surface roughness, morphology, topography, hardness, and the tribological behavior of shot peened materials has not yet been fully clarified [19,46,47,48]. 

Fundamentally being a mechanical surface treatment, shot peening is a complicated process, in which many parameters affect the process performance [20,49,50,51]. These can be classified into four main groups [52]: (1) shot characteristics (size [20,33,34,53,54,55], type [34,55], shape, density [34], hardness [34,53], and yield strength [20,33,34,52,53,54,56,57]); (2) process parameters [34] (shot flow rate, shot pressure [20,54,57,58], shot speed [59], impact angle [59,60], nozzle-to-target distance [60], nozzle diameter, and shot peening duration [20,32,60,61]); (3) target material properties (mechanical properties [34] such as hardness, yield strength, toughness, chemical composition and crystal structure [20,62]); and (4) environmental factors (temperature and humidity) [53,63]). Yildiran et al. reported that the surface roughness and hardness of Ti6Al4V alloy might significantly vary depending on shot size and peening pressure [5]. Another study [64] identified shot size and air pressure as the most effective parameters to improve surface roughness and hardness [64]. It is of vital importance to select a right combination of these parameters to obtain desired improvements in fatigue life of materials since some peening parameters such as shot size and pressure could significantly affect the microstructural and mechanical features of materials [19,26,33,34].

Numerous studies have investigated the fatigue life enhancement of Ti alloys (specifically Ti6Al4V) by performing shot peening with various parameters [24,43,65,66,67,68,69,70]. However, only a few studies have investigated the effects of shot peening and its parameters on the surface and subsurface properties of materials such as surface roughness, morphology, topography, and hardness in relation to their tribological behavior [34,44,71]. As shot peening inevitably modifies surface and subsurface properties as a function of peening parameters, it is important to elucidate the tribological behavior of shot-peened materials, which have not yet been fully understood in the literature [19,26]. Differing conclusions have been presented in the existing literature as some studies highlighted that shot peening improves the tribological behavior of materials [35,72,73], whereas others reported that shot peening detrimentally affects wear resistance [71]. The effect of shot peening on the friction and wear behavior of materials is still largely unexplored [26], and there has been an increasing interest on revealing the tribological behavior of shot peened materials [7,34,71,72,74,75,76].

There is a limited number of studies available in the existing literature focusing on understanding the influences of shot peening and shot peening parameters on the tribological behavior of Ti6Al4V alloy. Ganesh et al. [34] investigated the wear behavior of shot peened Ti6Al4V alloy and reported that the surface hardening occurred due to shot peening, improving the wear resistance of the alloy. Karaoglanli [22] investigated the wear performance of commercially pure (CP) Ti alloy shot peened at different Almen intensities and showed that the wear-volume loss was reduced with increasing Almen intensity as the surface hardness in relation to the Almen intensity has a significant role in the wear behavior. Similar findings were reported by Unal et al. [16] as they presented an improvement in the wear behavior of CP Ti (Grade 2) with shot peening. In contrast, Yang et al. [44] showed that shot peening detrimentally affects the fretting wear resistance of Ti6Al4V alloy during the early fretting wear period, while the wear rate is reduced in the long-term fretting wear process as the dominant wear mechanism changes from adhesion/peeling to delamination in both as-received and shot peened specimens [44]. Fridrici et al. [25] reported that shot peening does not have any significant influence on the fretting wear behavior of Ti6Al4V alloy compared to that of the polished specimen. 

In summary, although surface modifying processes (in particular, shot peening for the present paper) have great potential to improve the fatigue behavior of Ti alloys, there are limited studies on understanding how shot peening parameters influence (a) the surface and subsurface properties and (b) the tribological behavior of Ti alloy. The present study aims at investigating the effect of selected shot peening parameters (shot size and duration) on chosen properties of both surface and subsurface properties (hardness, microstructural features, coefficient of friction, mass loss, surface roughness, and surface morphology) of shot peened Ti6Al4V alloy.

## 2. Materials and Methods 

### 2.1. Preparation of Materials and Specimens 

Ti6Al4V alloy (grade 5) used in the experimental studies was purchased from TIMET (Titanium & Medical & Mining Company, Kocaeli, Turkey) in bar form (Ø20). Cylindrical samples were then cut (thickness: 10 mm) with a semi-automatic band saw (M42 27 × 0.9 × 3000). Before shot peening, a facing operation was performed to remove the saw marks on the sample surfaces by using a lathe. The samples were then ground with 320-, 600-, and 1200-mesh grits by an automatic grinding system to obtain a uniform and smooth surface topography before shot peening process. The composition of samples according to X-ray fluorescence spectrometer was as follows: Al: 5.629%, Fe: 0.089%, S: 0.006%, Si: 0.052%, Ti: 91.455%, and V: 2.769%.

### 2.2. Shot Peening Process

**Figure 1:**
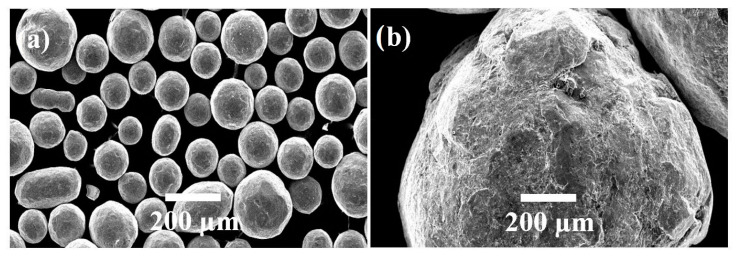
SEM images of stainless-steel shots used for peening: (**a**) smaller shot (S10) and (**b**) larger shot (S60) [37].

**Figure 2:**
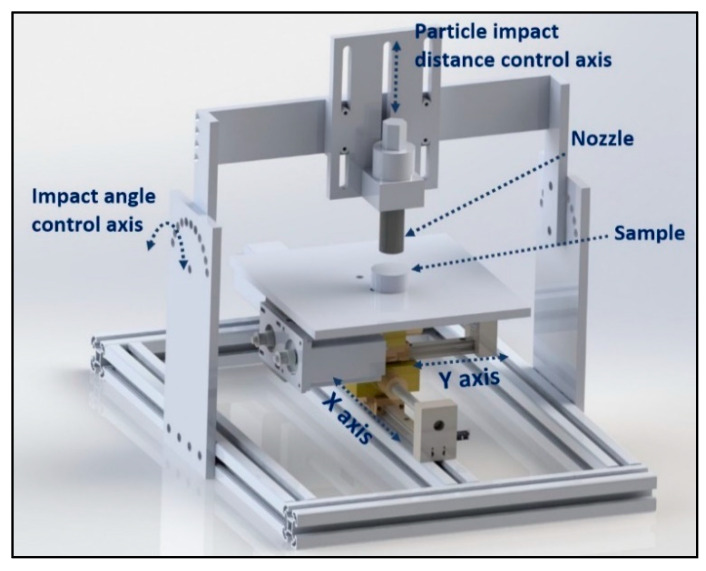
Shot peening system.

As detailed in the previous studies of the research team [5,37,39], shot peening was performed on the metallographically prepared Ti6Al4V alloy samples with two different sizes of stainless-steel shots (diameter ranges: 0.09–0.14 mm (type: S10) and 0.7–1.0 mm (type: S60)) (Figure 1) using the following parameters: peening duration of 5 min and 15 min; shot hardness of 450 HV; impingement pressure of 7 bar; and, impingement angle of 90°. After a preliminary characterisation of shot-peened samples for duration of 5 and 15 min, no important difference has been observed between samples (a) shot peened with S10 for 15 min and (b) shot peened with S10 for 5 min. Thus, the combination of S10 for 15 min has been excluded from the experimental plan. Shot peening was carried out in a specially designed CNC-controlled shot peening system (Figure 2) which includes an air compressor, a dehumidifier, a pressure regulator, a blasting cabinet along with other related pneumatic equipment such as valves and pipes. 

Shot peening was controlled by using Almen strips to check the process parameters and determine the reliability of the shot peening system. Almen strips were shot peened using the parameters above where spring heights were determined by using an Almen Gage with the following Almen intensities: S10 for 5 min—0.07 mmA, S60 for 5 min—0.20 mmA, and S60 for 15 min—0.36 mmA.

### 2.3. Subsurface Microstructural and Mechanical Characterisation

The cross-sectional microstructural and hardness examinations based on depth were carried out after the shot peening. The shot peened samples were cut by using a diamond cutting disc with a cutting device and then moulded in resin. The samples were ground (320-, 600-, 1200-, and 2000-mesh grits) and polished (3- and 1-micron diamond suspension with appropriate cloth). The polished samples were cleaned in an ultrasonic bath for 15 min with alcohol. For cross-sectional microstructural examinations, as-polished samples were etched (Kroll etch (2 mL HF, 4 mL HNO_3_, 92 mL H_2_O) [77]) for 5 s and were analysed using a scanning electron microscope (SEM) (Tescan Vega 2, Brno-Kohoutovice, Czechia). The effect of shot peening parameters on surface and subsurface hardness distribution depending on depth were measured (Zeiss micro Vickers hardness tester, Oberkochen, Germany) in HV_0.1_, 15 s, and five replicates.

### 2.4. Surface Characterisation

After the shot peening, the surfaces of the specimens were cleaned by ultrasonication in alcohol for 15 min. Then, the surface morphologies of the peened surfaces were investigated using an SEM equipped with an energy dispersive X-ray spectrometer (Tescan Vega 2, Brno-Kohoutovice, Czechia, with Oxford Instruments EDS detector, High Wycombe, UK). The effect of shot peening parameters on surface roughness and surface topography of Ti6Al4V alloy was measured with a surface profilometer (Bruker DektakXT, Karlsruhe, Germany). In the surface roughness analysis, an area of 1500 × 1500 µm^2^ was scanned from each sample surface, and the mean areal roughness value (S_a_) was determined before and after shot peening. 

### 2.5. Tribological Characterisation

Dry sliding wear tests were carried out using a ball-on-disk tribometer (Nanovea T50, Irvine, CA, USA) in the air at dry conditions via an alumina ball of 6 mm in diameter as counterpart using the following parameters: sliding speed: 0.05 m s^−1^, load: 5 N, distance: 100 m in accordance with the DIN 50 324 and ASTM G 99-95a standards. During the experiments, the variation of CoF with the distance was continuously recorded, and average CoF values were calculated from local CoF profiles. The worn specimens were ultrasonically cleaned in alcohol for 15 min. The wear tracks and the surfaces of counterparts were investigated via SEM-EDS. The change in the mass of the samples was measured via an electronic balance of accuracy of ±0.1 mg.

3D surface topography of the wear scars was observed by a surface profilometer (Bruker DektakXT, Coventry, UK), and the width and the depth of the wear scars (2400 × 2400 µm^2^) were determined. Finally, the worn specimens were cut for cross-sectional examinations and then prepared metallographically by using the same methodology in Section 2.3. The cross-sectional microstructural examinations of the worn specimens were analysed via SEM-EDS. 

## 3. Results and Discussion

### 3.1. Variation of Subsurface Microstructure and Hardness

**Figure 3:**
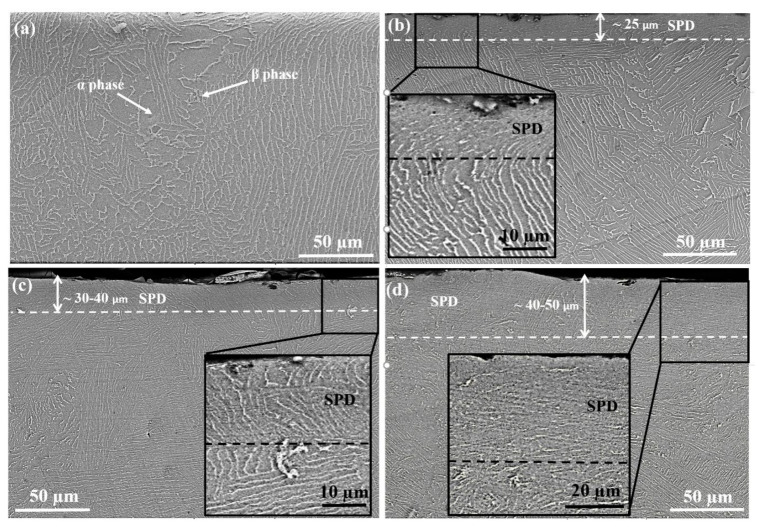
Cross-section microstructure examination of Ti6Al4V: (**a**) reference sample; (**b**) shot peened (S10, 5 min); (**c**) shot peened (S60, 5 min); (**d**) shot peened (S60, 15 min).

The cross-sectional microstructures were investigated (Figure 3) to understand the modifications in the surface and subsurface microstructure depending on the shot peening and shot peening parameters. The microstructure of the un-peened (reference) sample exhibited a homogeneous distribution of α and β phases (Figure 3a). Significant modifications in the near-subsurface microstructure of shot-peened samples were observable in comparison to the reference samples (inlet SEM images presented in Figure 3b–d. The near-surface microstructure at a depth of up to 25–50 µm was modified via shot peening due to severe plastic deformation (SPD). The microstructures within the SPD zones were different compared to that of the reference sample. It was observed that either the alpha + beta lamellar structure almost disappeared or the lamellar spacing was significantly reduced in the SPD zone, which could be attributed to the breakage of grain boundaries [78] due to the compression stresses caused by the shots [20]. The SPD region was deeper for large size shots (30 to 50 µm for S60 vs. approximately 25 mm for S10) and for longer peening times (from 30–40 µm for 5 min vs. to 40–50 µm for 15 min for S60). The mechanical properties of the alloy may be altered as the cross-sectional microstructure changes significantly by shot peening [79]. It is generally difficult to clearly observe the variation in crystal orientation, grain refinement, and lamellar spacing by SEM (which was also the case in the present study); thus, further studies such as those employing electron backscatter diffraction (EBSD) and field emission gun scanning electron microscopy (FEG-SEM) are required to justify the variation of crystal orientation and its effects on the surface and subsurface properties of shot peened Ti6Al4V alloy.

**Figure 4:**
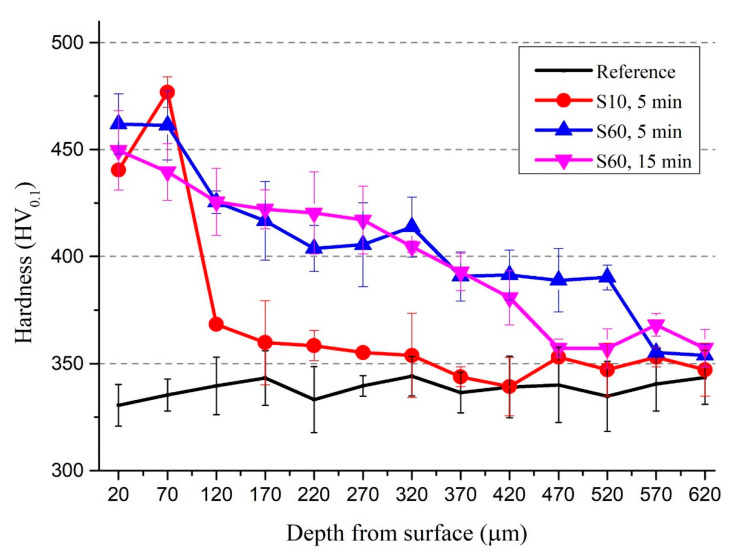
Cross-section hardness values of Ti6Al4V before and after shot peening with S10 and S60.

The hardness value of the reference sample varied in the range of 320–350 HV_0.1_ whereas the hardness of the peened samples increased up to 475 HV_0.1_ in areas close to the surface where it had the highest impact (Figure 4), i.e., the hardness improved up to approximately 35% as a result of surface modification compared to that of the reference sample. The hardness value decreased with the increase in depth for all process parameters, and after a certain depth (approximately 550 µm), the original hardness value of the material was measured and the affected are disappeared. The variation of hardness by surface modification (Figure 4) was in agreement with the previously discussed microstructural modifications (Figure 3). Namely, the increase in hardness was limited to approximately 120 µm depth for shot peening with S10 whereas the increase in hardness was more persistent and gradually declined with depth to background levels at approximately 550 µm for S60. The near-surface hardness of the samples processed with S10 had an increasing trend depth between 20–70 µm, whereas that of the samples modified with S60 had a slight decreasing trend in the SPD zone (Figure 4). This contrasting result is possibly because of the formation of undesirable microstructural defects such as micro-cracks and porosities due to the intensity of the plastic deformation caused by peening with S60 shots. It is known that the amount of plastic deformation during shot peening is closely related to the kinetic energy of particles [80] as a function of particle size and particle velocity/impingement pressure [78,79]. That being said, further characterisation may be needed to validate this explanation. 

The increase in hardness by shot peening has been previously explained through two hardening mechanisms which occur during shot peening: (1) grain refinement and twinning [7,29,81] and (2) plastic deformation [7,29,66,81,82]. The influences of both hardening mechanisms may vary depending on the microstructure and the composition of α and β phases in Ti6Al4V alloy [20]. In the present study, the cross-sectional SEM images showed that the microstructural features (α and β lamellar structure and lamellar spacing) significantly changed within the near-surface microstructure (depth down to 25 µm for S10, 30–50 µm for S60; Figure 3c,d), whereas the increases in hardness continued towards deeper layers (down to approximately 120 µm for S10, 550 µm for S60; Figure 4). An increasing shot size largely affected the microstructural features as well as the hardness (Figure 3 and Figure 4); however, the effect of peening time (5 min vs. 15 min for S60) was less important on changing the microstructural features and was insignificant in hardness improvement (Figure 4). As the amount of plastic deformation caused by shot peening is strongly dependent on the kinetic energy of particles [80], some studies report that higher kinetic energy can be achieved with large shots compared to that of the small shots [79,83]. To sum up, in the present study, the kinetic energy of the shots may not have been sufficient to deform the layers below approximately 120 µm depth for S10 shots and approximately 550 µm depth for S60 shots (Figure 4). Alikhani et al. [7] reported similar findings that deformation layer depth was limited since the needed energy for deformation of the underlying layers do not reach deeper regions. Similarly, although peening time increases the deformation density, the kinetic energy of shots may not be enough to modify deeper layers [50] as peening time seems even insignificant in hardness improvement (Figure 4).

### 3.2. Variation of Surface Morphology and Topography

Surface topography and morphology are important characteristics which affect the tribological behavior of materials [3,84,85]. It is stated that the surface roughness of shot peened materials depends on many factors, e.g., impingement pressure and impingement angle [5,59,86], shot size, and peening duration [5,87]. The present study focused on the effects of peening duration (5 and 15 min) and shot size (S10 and S60) while keeping the impingement angle and pressure steady.

**Figure 5:**
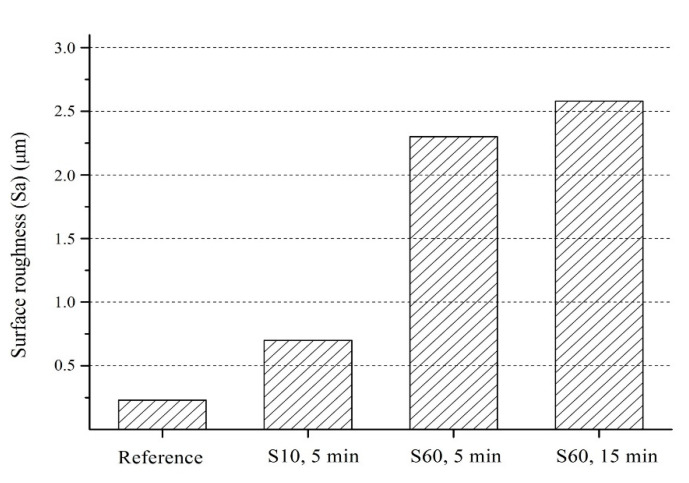
Areal surface roughness after shot peening with S10 and S60.

**Figure 6:**
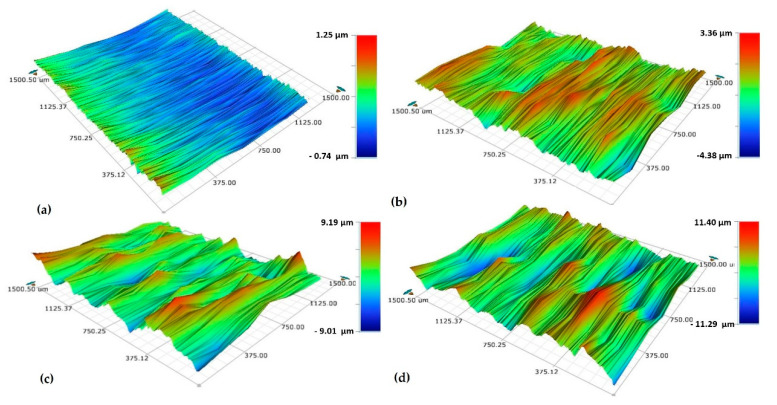
Surface topographies of Ti6Al4V; (**a**) reference sample, (**b**) shot peened (S10, 5 min), (**c**) shot peened (S60, 5 min), (**d**) shot peened (S60, 15 min).

A smooth surface was observed for the reference sample with a surface roughness (S_a_) of 0.22 μm (close to the mirror-polished surface [3]), while the samples shot peened with S10, S60 (for 5 min and 15 min) had much higher S_a_ (up to 2.55 μm) (Figure 5). Shot peening with S60 resulted in higher surface roughness than with S10 as the surface peened with S60 includes sharp, deeper valleys (blue contrast) and sharp, higher peaks (red contrast) (Figure 6c,d). The relative surface elevation varied between −9.01 and +9.19 µm at 5 min for S60 vs. between −4.38 and +3.36 µm at 5 min for S10 resulting in more smoothly shaped the surface with smaller valleys and peaks (Figure 6b). The shot size was the main parameter modifying the surface roughness (0.74 µm for S10 at 5 min vs. 2.27 µm for S60 at 5 min) having a major effect on the crater size formed on the surface during shot peening [87]. It has been stated that [39] larger particles have been responsible for higher surface hardness and roughness, and both properties fundamentally rely on the degree of the plastic deformation occurs on the peened surface [20,39]. 

Even though the increasing peening time does not change the kinetic energy of a single shot impacted on the surface during the peening, the total amount of introduced kinetic energy of the shots on the surface during shot peening goes up as the peening duration increases the coverage rate [88]. Thus, the maximum plastic deformation occurs on the surface, which affects the surface roughness, can be expected to be slightly controlled by the peening time. For the present study, the surface roughness slightly increased with higher peening times (2.27 µm for S60 at 5 min vs. 2.55 µm for S60 at 15 min). Zhang et al. [59] and Bagherifard et al. [87] have reported that the roughness rapidly increased at the first stage of the process and then changed into a steady state. They have explained this phenomenon in two stages; (1) roughness increases with the peening time with the formation of valleys and peaks on the relatively flat surface due to the initial impact of the shorts; and then, (2) the ratio of the increase of the roughness becomes almost zero since the following shots subsequently impact over the previously created peaks and valley, which only leads to the remodification of the surface [87].

**Figure 7:**
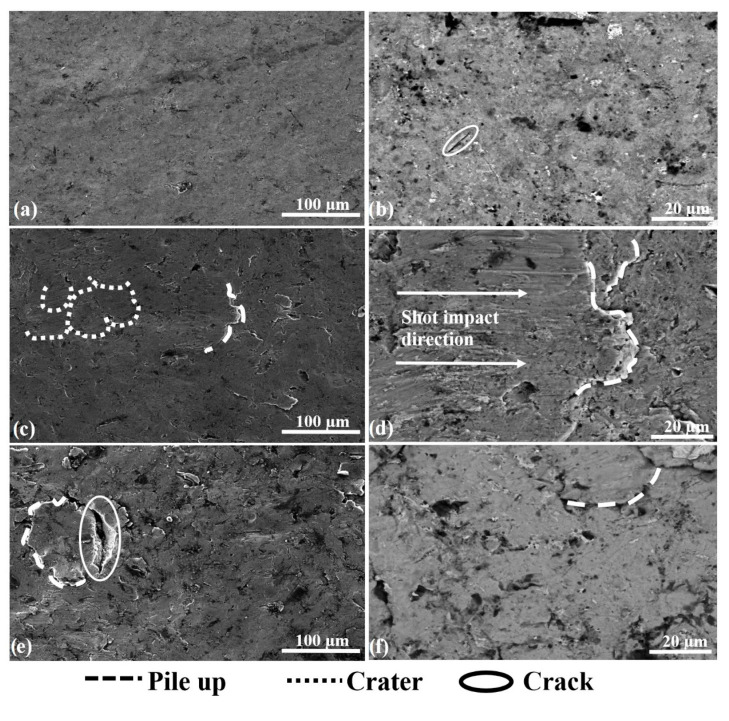
Surface morphologies at different magnifications after shot peening; (**a**) and (**b**) with S10, 5 min; (**c**) and (**d**) with S60, 5 min; (**e**) and (**f**) with S60, 15 min.

In order to better understand the variation of surface properties of the alloy depending on shot peening parameters, the surface morphology of the alloy at different magnifications after shot peening was investigated (Figure 7). Plastic deformation occurred on all surfaces after shot peening (Figure 7). The modifications of the shot peened surfaces under different parameters agreed with the modified subsurface microstructural features and surface properties previously discussed (Figure 3, Figure 5 and Figure 6). As each shot transfers its kinetic energy, plastic deformation on the sample surface may create craters. This mechanism repeats several times during the shot peening, which modifies the surface by forming peaks (characterised by high points of pile-up) and valleys (characterised by low points of craters) up to some extent [86,89,90]. Thus, the modification of the surface with plastic deformation could be considered the main reason for increased surface roughness [89,90,91]. Moreover, the larger shots cause higher plastic deformation, which leads to a more pronounced surface modification (Figure 7) accompanied by increases in roughness (Figure 5). These observations were in agreement with the previous work by the research team [37]. An adverse effect of shot peening with larger shots is SPD that may result with micro-cracks and delamination on the peened surfaces (Figure 7e), which is both undesirable and inevitable, observed in a similar fashion as reported in the literature [88,89,90]. Shot peening improves the mechanical material properties up to a limit, which mainly depends on the material’s strengthening capability [88,92]. After this point, increasing of peening time caused the remodification of the surface topography by overlapping following shots [60,93], commonly called as over peening effect which may cause the formation of microcracks and delamination on the surface [60,94] rather than improving mechanical properties [31,60]. To sum up, plastic deformation caused by peening initially forms valleys and peaks, which leads to an increase in surface roughness to some extent. In contrast, severe plastic deformation (SPD) causes surface and subsurface cracks, which are detrimental specifically for fatigue life, as highlighted in the existing literature.

### 3.3. Tribological Properties Depending on Shot Peening

**Figure 8:**
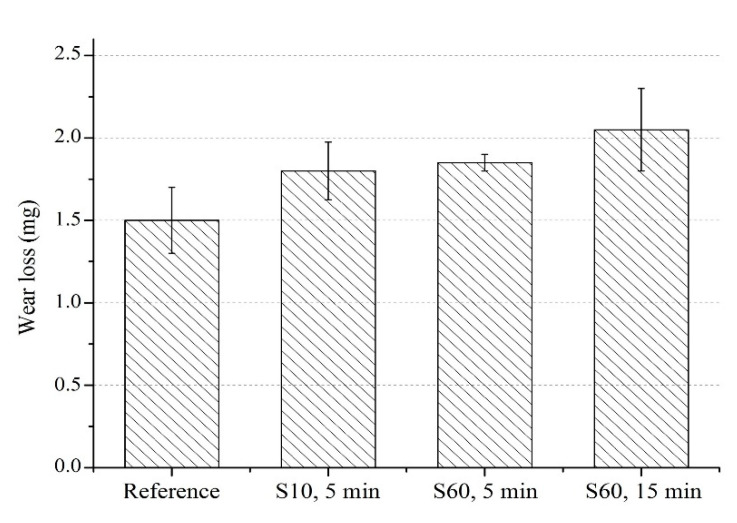
Wear loss after shot peening.

Wear is a critical industrial problem [73], which may lead to catastrophic failure before the estimated life of the material [36] due to the gradual degradation of surface and subsurface properties. Therefore, understanding the effects of surface treatments such as shot peening is important. Although it has been reported that there is no direct relationship between hardness and wear loss, it is generally expected that the increase in hardness leads to an improved wear resistance, which results in some decrease in mass loss [3,7,34,95]. The study samples had a slight mass loss (highest: 2.1 mg for S60 at 15 min) after the shot peening [33] (Figure 8), even though shot peening led to a significant increase in hardness of the surface and subsurface [71,81,82,96]. However, shot peening did not lead to a significant wear loss of Ti6Al4V alloy [25]. It should be underlined that the tribological behavior of the material [97], i.e., the interaction of two opposing surfaces, is strongly related to the surface roughness and integrity [44,97,98,99,100], which were dramatically changed by the shot peening due to SPD, as discussed in the previous sections (Figure 5, Figure 6 and Figure 7). Therefore, the CoF values and the worn surface morphologies of the shot peened specimens need to be explored in order to understand better the interaction of the asperities between the two rubbing surfaces and eventually the tribological behavior of the shot peened alloy.

**Figure 9:**
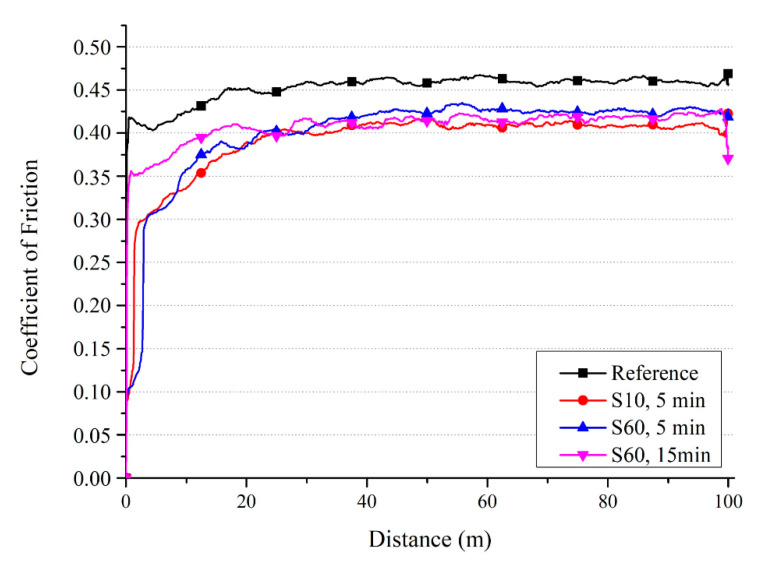
Coefficient of friction versus sliding distance for Ti6Al4V before and after shot peening.

CoF is a critical indicator affecting the tribological behavior of materials where smaller CoF corresponds to better wear resistance [3]. The variation of CoF versus sliding distance for the shot peened samples (Figure 9) showed a steep increase of CoF during the initial stage of the contact (specifically in the 0–5 m distance range) for all samples due to small contact per area, which exerts greater force [101], and then the CoF became stabilised with increasing distance. The CoF of Ti6Al4V alloy reference sample (approximately 0.46) was higher than that of the shot peened samples (approximately 0.40). The differences were small and varied to an extent on the shot peening parameters during the first 20 m and then became further less pronounceable with increasing distance (approximately 40 m). Similar findings have been previously reported on the fretting wear behaviuor of shot peened Ti alloys: Asperities formed by shot peening play an essential role on the CoF during the initial stage of the contact [25]. It is also expected that CoF may be significantly affected due to the modification of surface and subsurface microstructure and mechanical properties by shot peening [3,102]. Furthermore, it has been reported that the improvement in hardness and grain refinement generally reduces the CoF of materials [3,101]. 

**Figure 10:**
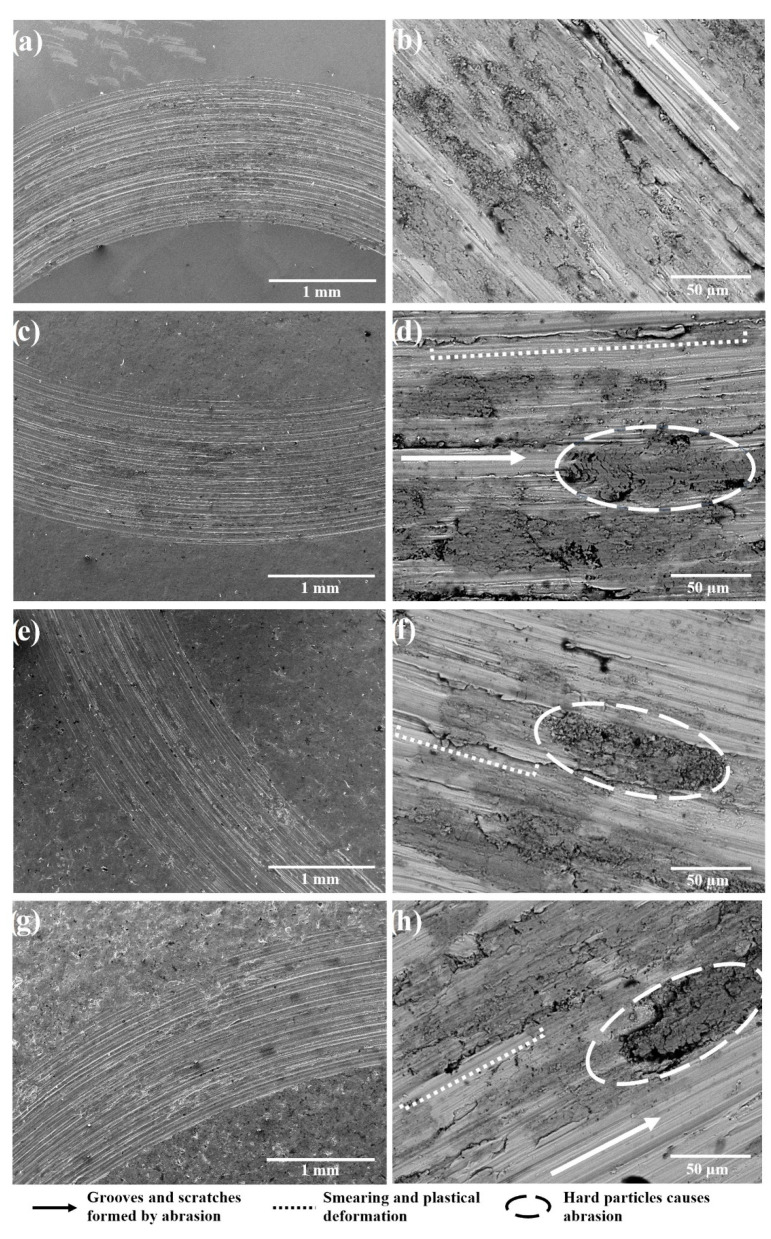
Surface morphologies of Ti6Al4V after wear test: (**a**) and (**b**) reference sample; (**c**) and (**d**) shot peened (S10, 5 min); (**e**) and (**f**) shot peened (S60, 5 min); (**g**) and (**h**) shot peened (S60, 15 min).

**Figure 11:**
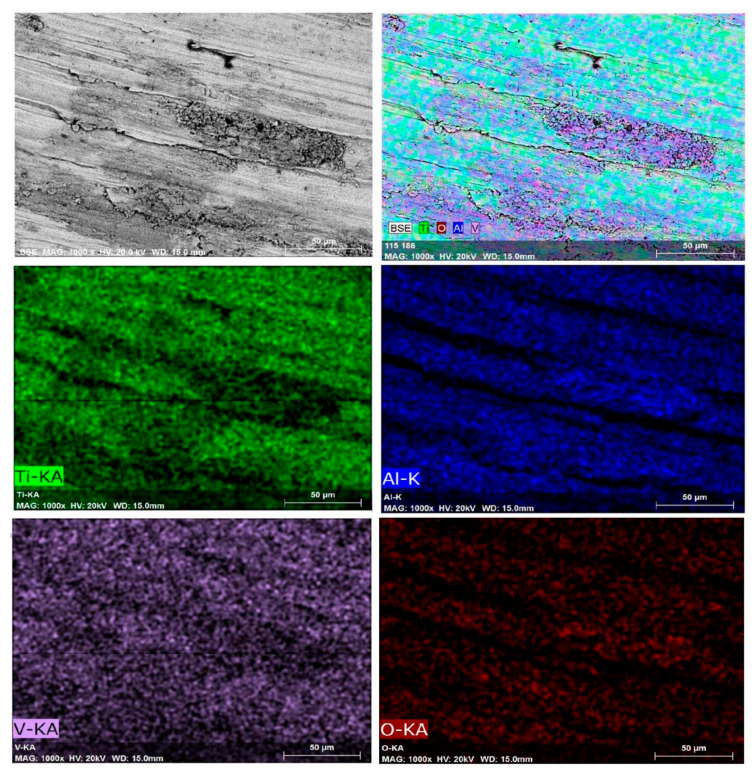
SEM-EDS mapping of shot peened surface after wear test (S60, 5 min).

**Figure 12:**
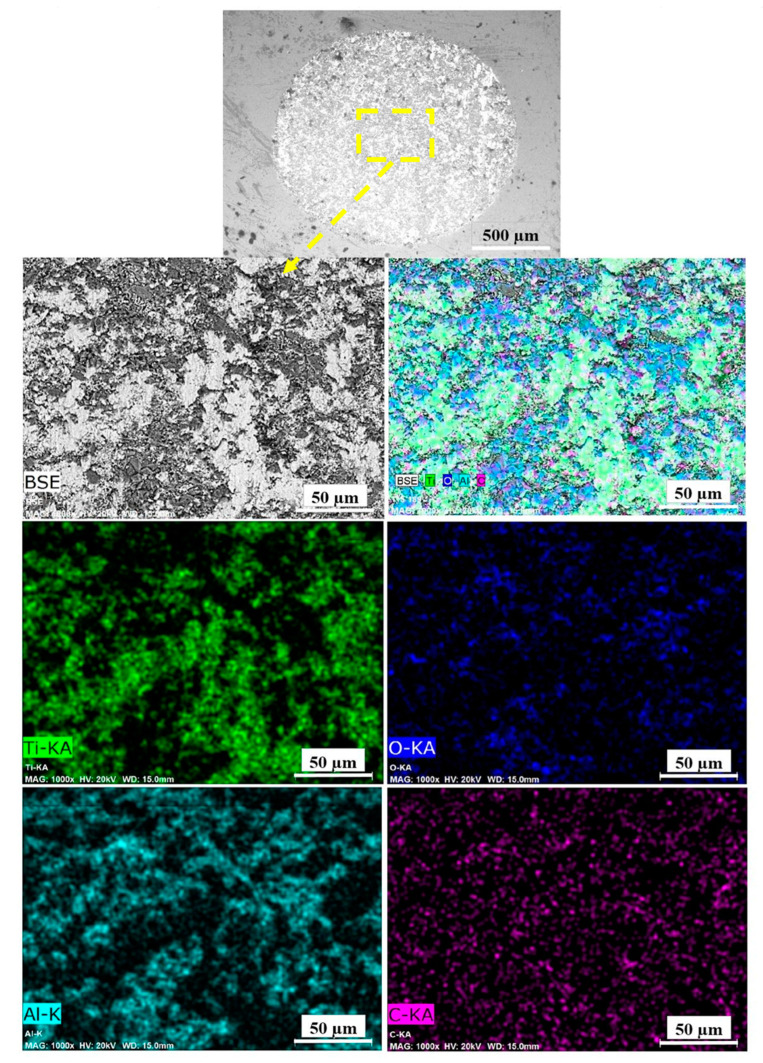
SEM-EDS mapping of alumina ball after wear test.

Stav et al. [84] proposed that the CoF of mechanical components subjected to reciprocal motion can be improved by shot peening. However, the change in surface morphology and topography by shot peening [97] may also affect the wear mechanisms and the contact pressure during wear [44,95,100]. Jain et al. [103] proposed that CoF is highly related with the surface finish as they presented that the CoF of bearing steel under dry sliding wear increases with increasing time due to decrease of surface roughness with the worn of asperities during contact. Wu et al. [104] reported that texturing surfaces of Ti6Al4V alloy by laser might decrease the CoF under sliding wear conditions (high-speed dry sliding test). On the contrary, Svahn et al. [98] stated that the rougher surface gave higher CoF with regards to their studies investigating the dry sliding wear behavior of bearing steel with different surface roughness. In conclusion, the underlying reasons of the variation of CoF of the alloy by shot peening can be related with following factors: (1) the changes in the microstructural and related mechanical features; and (2) the changes in the surface morphology and topography as a function of shot peening and peening parameters. The fluctuation of CoF (as observed in Figure 9) may occur due to the complex behavior of wear mechanisms during sliding [14]. Thus, it is crucial to understand which wear mechanisms dominantly affect the tribological behavior of shot peened Ti alloys as the next section investigates and compares the worn surface morphologies of the reference and shot peened samples in detail (Figure 10, Figure 11 and Figure 12). 

The worn surfaces of the reference and shot peened samples included regular scratches parallel to the wear direction (Figure 10). All wear tracks consisted of a material pile-up with smearing, formation, and breakdown of oxide layers (Figure 11); and, local grooves have been formed by micro-ploughing (Figure 10). The material pile-up via smearing (as in Figure 10) usually occurs with the plastic deformation due to the sliding contact of the ball [80]. The fragmentation of both the worn material and the oxide layers can occur during the dry sliding [105,106,107], which could behave like hard debris particles during the contact. Thus, the grooves on the worn surfaces may be formed with the three-body abrasion mechanisms caused by the abrasive actions of these hard debris particles [22,25,108]. The elemental mappings of Ti, Al, V, and O on the worn surface (Figure 11) indicates the formation of an unstable oxide layer on the wear track. The wear track includes oxygen-enriched debris particles (Figure 11), which may contribute to the three-body abrasion mechanisms as the presence of grooves (wear scars) indicates abrasion during sliding wear. Furthermore, the existence of Ti, Al, and V elements on the countersurface (alumina ball) indicates the material transfer occurred during the contact (Figure 12), which mainly corresponds to adhesion wear.

**Figure 13:**
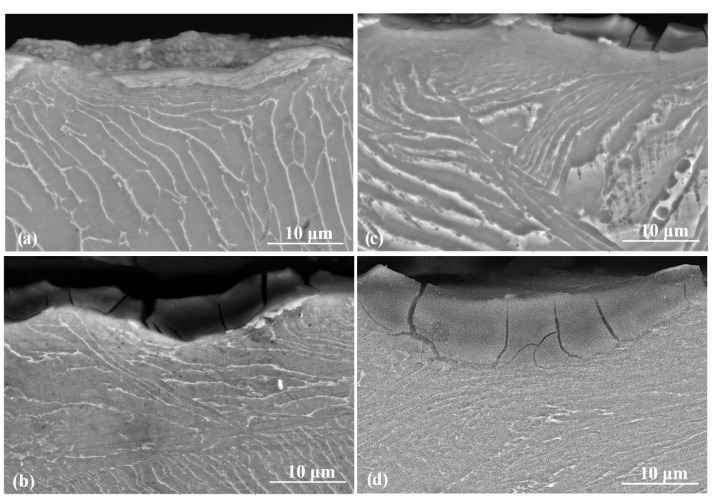
Cross-section microstructure examination after wear test; (**a**) reference sample; (**b**) shot peened (S10, 5 min); (**c**) shot peened (S60, 5 min); (**d**) shot peened (S60, 15 min).

**Figure 14:**
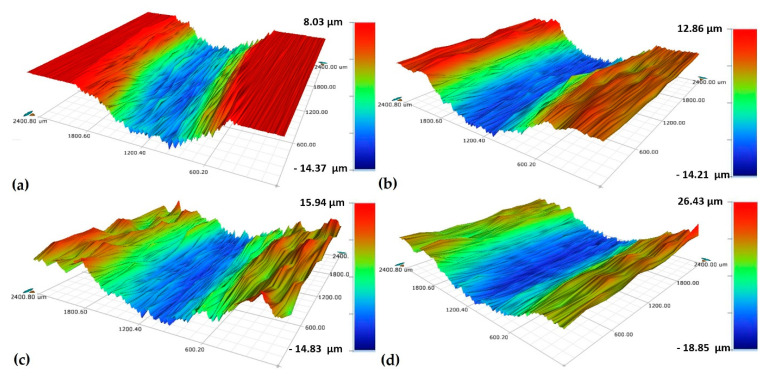
Three-dimensional (3D) surface topographies of worn specimens; (**a**) reference sample; (**b**) shot peened (S10, 5 min); (**c**) shot peened (S60, 5 min); (**d**) S shot peened (S60, 15 min).

As adhesion, abrasion, and oxide wear mechanisms have been generally reported in the literature on the tribological behavior of Ti and its alloys [3,80,105,108], it has been stated that the dominant wear mechanism changes with different mechanical surface treatments. These dominant wear mechanisms can be evaluated depending on two factors: (1) the modification of surface microstructure and mechanical properties; and, (2) tailored surface topography. Wen et al. [108] and Alikhani et al. [7] investigated the wear behavior of CP Ti before and after surface mechanical attrition treatment. Zhou et al. [3] studied the effects of laser peening (LP) on the dry sliding wear properties of Ti6Al4V alloy in Hank’s solution. These studies have reported that abrasive wear dominated the wear behavior after surface treatment due to the modification of subsurface microstructure and increased hardness. As a result, the surface treatments reduced the friction coefficient and the wear loss. Zhou et al. [3] have stated that after LP, the dominant wear mechanism changed to a slight adhesive and abrasive wear from oxidative wear with increasing surface hardness. On the contrary, Purcek et al. [105] have proposed that the wear of Ti and its alloys were controlled by oxidation wear; thus, the dry sliding wear resistance was not improved even though improved mechanical properties via equal-channel angular extrusion. Alikhani et al. [7] have reported that the formation and the size of the peaks and valleys after surface treatment were important for the wear behavior of materials since more profound valleys became inaccessible for the ball which contributed to the reduction of wear tracks. Amanov et al. [109] have reported that peaks wore off at the initial state of sliding rather than wearing off all surface homogeneously, which minimised CoF and wear loss due to the smaller contact area between the counter-surface and the worn surface [3]. In the present study, shot peening led to a slight increase in wear loss and decrease in CoF, and similar wear mechanisms occurred at all samples. Roughened and oxidised surfaces by shot peening mainly affected the wear behavior of the shot peened samples in the initial stages of wear (first 20 m, Figure 9). The modification of the surface topography and subsurface microstructure along with the improvement of hardness resulted in smaller CoF compare to that of the reference sample. However, the applied higher pressure due to the small contact area on the peak points caused the fragmentation of the modified surface topography and lead to easy removal of material and a slightly higher wear loss after shot peening, which is highlighted via SEM-EDS (Figure 10, Figure 11 and Figure 12). The 3D surface topographies along with the depth of wear tracks and the subsurface microstructure of worn samples (Figure 13 and Figure 14) were interpreted in the following section to clarify the effects of shot peening on the tribological behavior of Ti alloys. 

Microstructural changes were observed in surface and subsurface regions of even the reference sample due to the wear-induced plastic deformation (Figure 13a) [105]. The region beneath the surface of the wear track of the reference sample significantly changed during the wear process, probably due to the work-hardening and shear strain on the surface [7] (Figure 13a). The cross-sectional image of the wear tracks of shot peened sample with S10 (Figure 13b), similar to the reference sample (Figure 13a) showed the formation of wear marks, the presence of the oxide layer, and microstructural change occurring in the wear affected area. A slight material pile up on the edges of the wear track can be seen in the worn surface topography of the reference sample [76] (Figure 14a), which highlights the plastic deformation taken place during the dry sliding conditions [3,7,80]. This agreed well with the cross-sectional SEM results of the wear tracks since even the subsurface microstructure of the reference sample was modified due to contact during dry sliding tests (Figure 13a) [7]. It is clear that the modified subsurface microstructure by shot peening plays an important role on the tribological behavior of the alloy as the modified region was clearly affected by the wear tests [3,7,80]. 

**Figure 15:**
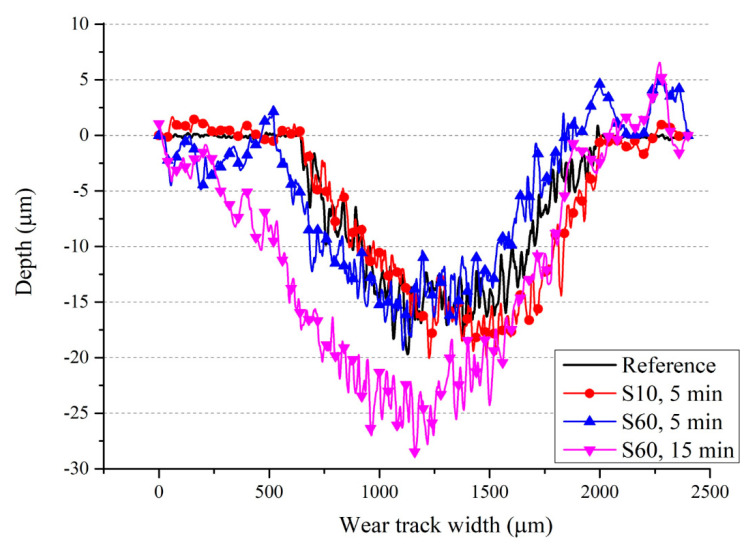
Three-dimensional (3D) surface topographies of worn specimens: (**a**) reference sample; (**b**) shot peened (S10, 5 min); (**c**) shot peened (S60, 5 min); (**d**) shot peened (S60, 15 min).

The depth of the wear track rises to 30 µm under severe shot peening conditions (Figure 15), which correlates with increases in mass loss with shot peening as previously discussed (Figure 8). The width of the wear tracks of shot peened samples is larger than that of the reference sample (Figure 14 and Figure 15). The presence of deep valleys and pits formed on the surface of the samples shot peened with S60 for 15 min are higher compared to that of the reference sample and sample shot peened with S10 (Figure 14 and Figure 15). Hence, a more severe wear process occurs with shot peening which can be attributed to the changed surface integrity (morphology and roughness) and microstructure by shot peening [7]. The roughened surface morphology formed by shot peening seems to be easily worn off under dry sliding conditions, whereas the reference sample shows a more stable wear regime. This partly explains the variation of CoF depending on shot peening and peening parameters (Figure 9).

## 4. Conclusions

The present study investigated the variation of surface and subsurface properties (microstructural, mechanical, surface, and tribological) of Ti6Al4V alloy depending on shot peening and its parameters (shot size and peening time). In summary, shot size significantly affected the microstructural features along with hardness, whereas peening time had limited impact. Surface hardness improved up to approximately 35% by shot peening compared to that of the un-peened (reference) sample due to severe plastic deformation, and the variation of hardness agreed with the modification of the microstructural features. Considering the relatively weak tribological properties of this alloy, improving hardness via shot peening would be of high importance in both aerospace and biomedical applications, specifically when the alloy is to be used under friction. In summary, shot peening led to an increase in wear loss (maximum 25%) and a decrease in coefficient of friction (CoF) (maximum 12%) as similar wear mechanisms occurred at all samples. 

Surface roughness plays a particular role on the biological response (e.g., interfacial stability and osseointegration) of titanium alloys; and, the present study reports crucial findings on how surface features could be tailored via shot peening depending on peening parameters. In more detail, shot peening with larger shots led to a more pronounced surface modification accompanied by increases in roughness (average surface roughness being three times of that of the surface peened with smaller shots) due to higher plastic deformation, indicating the shot size as the main parameter modifying roughness. By contrast, a higher peening time increased roughness only approximately 12%, while at the same time longer peening duration with large shots led to an unwanted formation of micro-cracks and delamination on the peened surfaces, which needs to be limited to preserve the surface integrity of the alloy in biomedical and aerospace applications. In the present study, shot peening improved the mechanical properties up to a degree; and after a certain point, increasing peening time caused the remodification of the surface topography by overlapping following shots commonly called as over peening effect, which may cause the formation of microcracks and delamination on the surface rather than improving mechanical properties. Thus, it can be suggested that shorter peening times (up to 5 min) with larger shots (e.g., S60) would be the appropriate option to obtain better mechanical properties with less compromise in surface roughness. For future applications wanting to employ these parameters, to address their case-specific needs, we recommend further optimisation studies that will help obtain tailored parameters that will yield the best results.

On the one hand, the modification of the surface features and subsurface microstructure along with the improved hardness resulted in smaller CoF compared to that of the reference sample. On the other hand, the increase of contact pressure due to the small contact area led to easy removal of the peaks formed during shot peening; and thus, a slightly higher wear loss. The present study elucidates the surface, subsurface and tribological response of Ti6Al4V alloy shot peened under different parameters (shot size and peening time), which is a matter of great importance for understanding and improving the relatively low wear resistance of the alloy. The obtained results would be specifically useful for the processing of gears and bars in the field of automotive, landing gears and turbine blades in the field of aerospace, and hip implants in the field of biomaterials where fatigue life, surface properties and tribological features are of great importance.

Future studies are recommended including (1) tribological investigations such as dry- and lubricated-sliding wear tests for longer distances to understand further how modified surface and subsurface features via shot peening affect the CoF and wear rates of the alloy; and, (2) work to reveal the influence of shot peening parameters on the microstructural features such as crystallographic orientation, grain refinement, lamellar spacing and dislocation density supported by advanced microstructural characterisation (electron backscatter diffraction and high-resolution transmission electron microscopy).
